# Supplementary material for: MetaRibo-Seq measures translation in microbiomes
Source: Nat Commun. 2020 Jun 29;11:3268. doi: 10.1038/s41467-020-17081-z (PMC7324362; doi:10.1038/s41467-020-17081-z)
Supplement: Supplementary file 10 — Supplementary Data 7 [file 41467_2020_17081_MOESM10_ESM.zip › File2/Confidence_High_Taxonomy/345536_out.krona.html]

Javascript must be enabled to view this page.

members
magnitude
magnitudeUnassigned
count
unassigned
taxon
rank

345536\_out

6

superkingdom
2
6

6
976
phylum

class
6
200643

6
171549
order

6
1853231
family

genus
283168
6

6
28118
species

SRS011134\_contig\_number\_contig-100\_2682.246836SRS022609\_contig\_number\_44172SRS049773\_contig\_number\_contig-100\_722.207064SRS077194\_contig\_number\_contig-100\_1574.189710SRS1041129\_contig\_number\_contig-100\_2983.76323SRS1055022\_contig\_number\_contig-100\_1562.114087
